# The Role of Long Non-Coding RNAs in Human Endoderm Differentiation

**DOI:** 10.3390/ncrna11020029

**Published:** 2025-04-13

**Authors:** Annanda Lyra Ribeiro, Bruno Dallagiovanna

**Affiliations:** Stem Cells Basic Biology Laboratory, Carlos Chagas Institute—FIOCRUZ/PR, Curitiba 81350-010, Brazil; annandalyrar@gmail.com

**Keywords:** lncRNAs, human endoderm differentiation, stem cells

## Abstract

The human genome sequencing revealed a vast complexity of transcripts, with over 80% of the genome being transcribed into non-coding RNAs. In particular, long non-coding RNAs (lncRNAs) have emerged as critical regulators of various cellular processes, including embryonic development and stem cell differentiation. Despite extensive efforts to identify and characterize lncRNAs, defining their mechanisms of action in state-specific cellular contexts remains a significant challenge. Only recently has the involvement of lncRNAs in human endoderm differentiation of pluripotent stem cells begun to be addressed, creating an opportunity to explore the mechanisms by which lncRNAs exert their functions in germ layer formation, lineage specification, and commitment. This review summarizes current findings on the roles of lncRNAs in endoderm differentiation, highlighting the functional mechanisms and regulatory aspects underlying their involvement in cell fate decisions leading to endoderm development. The key lncRNAs implicated in endoderm differentiation are discussed, along with their interaction with transcription factors and RNA-binding proteins and modulation of signaling pathways essential for endoderm development. Gaining insight into the regulatory roles of lncRNAs in endoderm differentiation enhances the understanding of developmental biology and provides a foundation for discovering novel lncRNAs involved in cell fate determination.

## 1. Introduction

In the early years of the twenty-first century, the successful sequencing of the human genome revealed its 3 billion base pairs of DNA, including extensive non-coding regions [1]. The discovery that over 80% of the genome is pervasively transcribed into RNAs that do not encode proteins, the so-called non-coding RNAs (ncRNAs), challenged the idea that protein-coding genes are the sole regulators of gene expression and cellular processes [2]. Recent studies demonstrated that, combined with proteins, ncRNAs play key roles in stem cell differentiation [3] and embryonic development [4], as well as in the progression of diseases such as cancer [5] and neurodegenerative disorders [6], reinforcing their importance in different physiological states.

Long non-coding RNAs (lncRNAs) are among the ncRNA family’s most abundant and functionally diverse classes. Current estimates based on high-throughput RNA sequencing and comprehensive annotation projects, such as LNCpedia and ENCODE, indicate the existence of more than 16,000 lncRNAs in the human genome [7], most of which still have no assigned biological function [8]. lncRNAs are classified as transcripts longer than 200 nucleotides with limited or no protein-coding potential [7], but recent literature is adopting a minimum length of ≥500 nucleotides to define and better distinguish them from other types of ncRNAs, such as micro-RNAs (miRNAs) [9]. Like messenger RNAs (mRNAs), lncRNAs may undergo polyadenylation, capping, and alternative splicing but are less evolutionary conserved and typically exhibit low expression levels across cell types [9,10]. Nonetheless, lncRNAs demonstrate high cell type and tissue specificity, implying a potential role in fine-tuning cell type-specific processes [10,11].

A definitive and universally accepted classification that includes all lncRNAs has not yet been established, although several criteria have been proposed to classify them based on different characteristics and functions [12]. lncRNAs can be classified into five categories according to their genomic origin: intergenic, intronic, sense, antisense, and bidirectional [13]. Intergenic lncRNAs originate between two protein-coding genes, whereas intronic lncRNAs are transcribed from within long introns of coding sequences [14]. Sense lncRNAs are synthesized from the same strand and direction as their associated coding gene and may overlap with exons, introns, and untranslated regions (UTRs) of the gene [14]. Antisense lncRNAs, on the other hand, are transcribed in the opposite direction of protein-coding genes [14]. In the case of bidirectional lncRNAs, the transcription starts close to the promoter of a protein-coding gene but in the opposite direction [15].

Another method for classifying lncRNAs is based on their function, grouping them according to how they interact with DNA, RNA, and proteins to regulate gene expression at different levels, including transcriptional, post-transcriptional, and epigenetic regulation [13]. Signal lncRNAs act as molecular signals that regulate transcription in response to specific cellular cues or environmental stimuli [7]. Xist is a classic example of signal lncRNA, as its expression dictates X chromosome inactivation in females by inducing DNA methylation and histone modifications [16]. lncRNAs can also function as molecular sponges for transcription factors (TFs), RNA-binding proteins (RBPs), and miRNAs, effectively sequestering these molecules and preventing their interaction with target genes [15]. For example, lncRNA MALAT1 acts as a sponge for miR-145, sequestering it and preventing its tumor-suppressive role in targeting oncogenes, thereby enhancing tumor growth and metastasis in various types of cancer [17,18]. Additionally, lncRNAs may act as guides, directing regulatory protein complexes—such as ribonucleoprotein (RNP) complexes—to specific target genes [15]. FENDRR, for instance, is a guide lncRNA that recruits Polycomb repressive complex 2 (PRC2) to the proximity of genes involved in lateral mesoderm formation, such as FOXF1 [19].

Moreover, lncRNAs can assume structural functions in gene regulation in two distinct ways: as scaffolds [20] or architectural elements [21]. Transcripts classified as scaffold lncRNAs serve as structural platforms for assembling RNP complexes and other multi-component RNA-protein assemblies [7,20]. Whether an assembled RNP complex activates or represses transcription is dependent on the proteins and RNAs contained within the complex [22,23]. One example is lncRNA TERC, which acts as a scaffold for the telomerase complex, enabling the addition of telomeric repeats and maintaining telomere length [24]. Architectural lncRNAs, on the other hand, function as scaffolds for nuclear bodies [25]. Of the five architectural lncRNAs described so far, NEAT1 is the most well-studied example involved in forming paraspeckles [25]. NEAT1 encodes two isoforms, NEAT1_1 and NEAT1_2, with the latter being essential for assembling RBPs and heterogeneous nuclear ribonucleoproteins (hnRNPs) required for paraspeckle formation. Without *NEAT1* transcripts, stable paraspeckle formation is blocked [26].

Given the diverse mechanisms by which lncRNAs exert their functions, accurately predicting the behavior of novel lncRNAs in state-specific cellular conditions remains a challenge, particularly when relying on data from non-human models [27]. In this context, human pluripotent stem cells (hPSCs) provide a controlled environment for studying lncRNA expression and function, mimicking early developmental processes such as pluripotency maintenance, germ layer formation, and cell differentiation [27,28]. A few lncRNAs have been reported to play pivotal roles in both pluripotency maintenance and the differentiation of PSCs into the three germ layers—mesoderm, endoderm, and ectoderm—during the early stages of embryogenesis. For instance, lincRNA-RoR was the first functional lncRNA reported to promote reprogramming of human induced pluripotent stem cells (hiPSCs) and maintenance of pluripotency in human embryonic stem cells (hESCs) [29], functioning as a competitive endogenous RNA (ceRNA) that sequesters core pluripotency factors such as OCT4, SOX2, and NANOG [30]. Similarly, lncRNA TUNA promotes the formation of RNA multiprotein complexes at the promoters of the aforementioned pluripotency factors, activating their transcription and sustaining the pluripotent state of mouse embryonic stem cells (mESCs) [31]. As differentiation progresses, lineage-specific lncRNAs become regulators of germ layer specification. lncRNA yylncT, for example, localizes at the Brachyury (T) gene locus and regulates the TGF-β signaling pathway, indispensable for mesoderm differentiation [32]. lncR492, conversely, acts as a lineage-specific inhibitor of neuroectodermal differentiation by interacting with the HuR protein [33].

However, the role of lncRNAs in human endoderm differentiation remains largely unexplored and is still being actively investigated. This review highlights the emerging functions of lncRNAs in definitive endoderm differentiation, emphasizing their mechanisms of action and regulatory networks. It further examines the identification and characterization of endoderm-associated lncRNAs, highlighting their interactions with TFs, RBPs, and key signaling pathways that regulate endoderm commitment. By integrating recent findings, this review aims to provide a comprehensive perspective on the contribution of lncRNAs to endoderm differentiation and their broader implications in developmental biology.

### Regulation of Human Endoderm Differentiation

In mammals, gastrulation marks a critical stage in embryonic development, during which totipotent epiblast cells undergo specification of the three germ layers—mesoderm, endoderm, and ectoderm [34]. Each germ layer establishes the foundation for distinct tissues and organs, with the endoderm serving as the precursor to the epithelial lining of the digestive and respiratory tract and organs such as the liver, pancreas, thyroid, stomach, and bladder [35,36]. Not surprisingly, endodermal tissues take part in different homeostatic process, including glucose metabolism and nutrient absorption [36], and their disruption has been linked to several diseases such as diabetes, fatty liver disease, and cancer [37].

The ethical and technical limitations associated with using human embryonic tissues initially hindered insights into the transcriptional regulation of endoderm differentiation [36,37], until the emergence of hPSCs and iPSCs-based in vitro differentiation models offered a new approach to explore the regulatory network behind this process [36]. Since then, key signaling pathway and metabolic and epigenetic factors have been identified as regulators of endoderm differentiation [38]. Four main signaling pathways have been implicated in endoderm differentiation of hESCs: Nodal/Activin A, BMP, WNT, and FGF [39]. The formation of the primitive streak (PS) relies on the coordinated activity of Nodal/Activin A, WNT, BMP, and FGF, as blocking any of these pathways disrupts PS development and impairs endoderm differentiation [40,41]. Once PS is established, endoderm specification is driven by high Nodal/Activin A and low endogenous FGF signaling [37]. Definitive endoderm differentiation is achieved when Nodal/Activin A activates TGF-β receptors responsible for phosphorylation of SMAD2/3 [42]. The phosphorylated SMAD2/3 complex translocates to the nucleus, where it activates the expression of key endoderm transcription factors, such as FOXA2, SOX17, GSC, and GATA6, promoting endoderm differentiation [42]. A more detailed overview of the regulation of human endoderm differentiation is available in specialized review articles [34,35,36,37,39,43].

## 2. Long Non-Coding RNAs in Human Endoderm Differentiation

The potential roles and mechanisms by which lncRNAs may influence definitive endoderm differentiation remained unexplored for a long time [44]. While epigenetic and TFs regulation were already known to contribute to endoderm formation [45], the first lncRNA linked to this process was identified less than a decade ago, when DEANR1 was shown to be a transcriptional activator of FOXA2 [46]. DEANR1 facilitates endoderm differentiation by interacting and recruiting SMAD2/3 to the promoter region of FOXA2 [46]. Recently, translated microproteins from DEANR1 were identified in pancreatic endocrine differentiation, but whether its translational potential also applies to endoderm differentiation remains unclear [47]. The following year, lncRNA DIGIT was reported to be divergently transcribed from the GSC locus, where it regulates GSC expression and promotes endoderm differentiation in mESCs and hESCs [48]. Further studies revealed that DIGIT interacts with the terminal domain of BRD3, promoting the formation of phase-separated condensates of proteins. These condensates enhance BRD3 binding to acetylated H3K18-enriched regions during endoderm differentiation, facilitating transcription [49]. lncRNA Gas5, on the other hand, has been suggested to inhibit endoderm differentiation in mESCs, possibly through its interaction with TFs and DNA methylation regulators, which may repress endoderm-specific gene expression while promoting the maintenance of pluripotency [50]. The inhibition of endoderm differentiation was also reported for ANCR. In this case, lncRNA ANCR interacts with the RBP PTBP1 to enhance the stabilization of ID2 mRNA, thereby restricting the differentiation of human adipose tissue-derived mesenchymal stem cell differentiation towards endoderm [51].

More recently, GATA6-AS1 was identified as an essential lncRNA for endoderm differentiation by modulating TGF-β ligand binding to SMAD2/3 receptors, activating GATA6 transcription [44]. GATA6-AS1 positively correlates with GATA6, and its deficiency is sufficient to downregulate the endoderm gene program [44]. LINC00458 was also reported to interact with SMAD2/3 in the nucleus to regulate substrate-induced endoderm lineage specification through control of matrix stiffness [52]. The authors demonstrated that LINC00458, along with other lncRNAs, is upregulated in soft substrates, highlighting the role of mechanical cues in the endoderm differentiation of hPSCs [52]. In 2021, the lncRNAs HOXA-AS3 and HOXB-AS3, transcribed from the central regions of the HOXA and HOXB clusters, were shown to modulate chromatin accessibility during endoderm lineage commitment [53]. Two years later, the desert lncRNA HIDEN was found to physically interact with IMP1 to facilitate mRNA stabilization of Frizzled 5 (FZD5) receptors, leading to the activation of the WNT signaling pathway and the consequent regulation of endoderm differentiation [54]. HIDEN was the first endodermal “desert” lncRNA discovered [54], and its mechanism of action is detailed in the following section. The most recently identified endodermal lncRNA, TREX-17, is transcribed within the same topological domain as SOX17, another important TF involved in definitive endoderm formation [55]. Table 1 summarizes the lncRNAs currently identified in endoderm differentiation and their assigned mechanisms of action in this germ layer.

### 2.1. GATA6-AS1

GATA6 antisense RNA 1 (GATA6-AS1) is an lncRNA divergently transcribed from the GATA6 locus [44], which encodes one of the six TFs that constitute the GATA family [56]. This gene family is composed of TFs with two highly conserved zinc finger DNA-binding proteins that recognize the (A/T)GATA(A/G) consensus nucleotide sequence [56] and is widely known for its participation in embryonic development and germ layer differentiation [57]. GATA6, in particular, has been considered essential for endoderm differentiation and lineage specification of endoderm-derived organs and cells, such as the pancreas and their residing β cells [58]. Since GATA6-AS1 is genomically adjacent to GATA6 and antisense lncRNAs were reported to have similar expression patterns and regulatory roles to their neighboring protein-coding genes [59], the potential role of GATA6-AS1 in endoderm differentiation began to be addressed [44].

**Figure 1:**
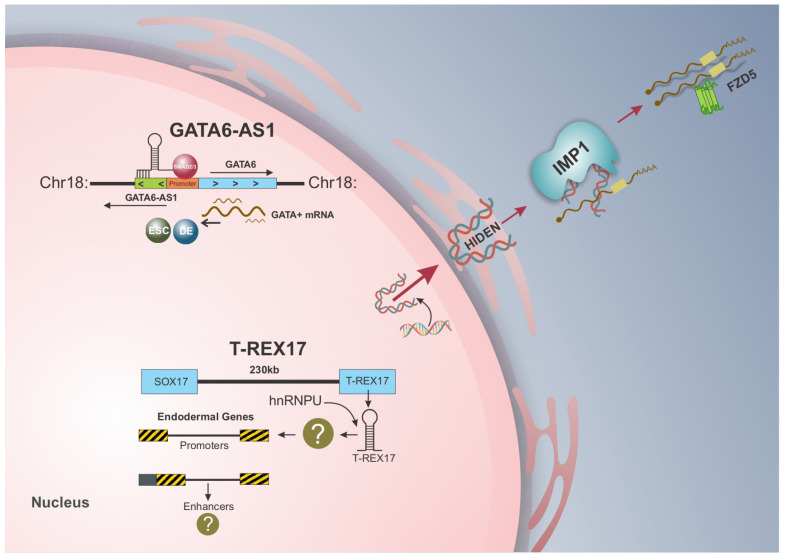
lncRNAs and their mechanism of action in endoderm differentiation of hiPSCs. GATA6-AS1 and T-REX17 are expressed in the nucleus of endoderm cells, while HIDEN is found in the cytoplasm. With different mechanisms of action, GATA6-AS1 functions in *cis* during endoderm differentiation by facilitating SMAD2/3 recruiting and binding to the GATA6 promoter, leading to its activation. T-REX17, on the other hand, is transcribed with the same topologically associated domain as SOX17 but does not regulate this transcription factor, implying a trans-acting role in differentiation through its interaction with heterogeneous ribonucleoprotein (hnRNP), particularly hnRNPU. HIDEN, first desert lncRNA associated with endoderm differentiation, physically interacts with IMP1 protein and enhances the stability of FZD5 by mediating the interaction between IMP1 and FZD5 mRNA.

Antisense lncRNAs regulate gene expression through two main mechanisms: *trans* and *cis*-regulation [15]. While *trans*-acting antisense lncRNAs regulate the expression of genes far located from their transcription site by interacting with other regulatory regions within the cell, *cis*-acting antisense lncRNAs interact with perfect sequence complementarity to the promoter region of their originating gene, modulating the transcriptional activity of neighboring transcripts [13]. In endoderm differentiation, GATA6-AS1 is upregulated in a timeline similar to GATA6. Notably, the knockdown of GATA6-AS1 led to a decrease in GATA6 expression, suggesting that GATA6-AS1 functions in *cis* during endoderm differentiation by facilitating SMAD2/3 recruitment and binding to the GATA6 promoter, activating its transcription [44] (Figure 1).

Based on this role, it could be suggested that GATA6-AS1 functions as a scaffold lncRNA, providing a structural platform for the recruitment and assembly of regulatory molecules at specific genomic locations [60]. Interestingly, Kuo and colleagues [61] demonstrated through computational methods that GATA6-AS1 may form RNA–DNA triple helices within the GATA6 promoter region during cardiac development, providing space for physical interactions with other molecules in this region. Although this mechanism was proposed in the context of cardiac differentiation, this finding raised the hypothesis that the formation of RNA–DNA triple helices within the GATA6-AS1 gene body could be the facilitating platform for the recruitment and binding of SMAD2/3 in endoderm differentiation [44]. Further experimental validation is necessary to support the computationally predicted mechanism for each differentiation model, especially endoderm.

At the cellular level, hESCs depleted from GATA6-AS1 exhibited severe impairment in endoderm differentiation. However, GATA6 overexpression could rescue the endoderm defects caused by GATA6-AS1 depletion, indicating that GATA6-AS1 is necessary for GATA6 transcriptional activation by SMAD2/3 [44]. Interestingly, the TGF-β/SMAD signaling pathway is at the core of endoderm commitment [27]. Once activated by TGF-β ligands, such as Activin/Nodal, SMAD2/3 are phosphorylated and form a complex with SMAD4, which then translocates to the nucleus. There, the SMAD complex binds to regulatory elements within the promoter regions of endoderm-specific genes and collaborates with TFs, chromatin remodelers, and other molecules to drive differentiation [44]. In addition to GATA6-AS1, other lncRNAs have already been reported to modulate endodermal lineage specification by regulating SMAD2/3 in hiPSCs [46,52], suggesting that lncRNAs may play a unique role in fine-tuning SMAD functions in pluripotency exit and differentiation [44].

The coordinated expression between GATA6 and GATA6-AS1 is not restricted to endoderm differentiation and has been observed in different tissues, including the colon, ovary, and placenta [57], as well as in other cellular processes, such as cardiac development in hiPSCs [57,61]. GATA6-AS1 also acts in a *cis* manner during cardiac differentiation as its knockdown downregulated not only GATA6 expression but also WNT targets and signaling genes, impairing cardiomyocyte formation [57]. The WNT/β-catenin signaling pathway is activated during heart development by GATA transcription factors, which help integrate canonical and non-canonical pathways to balance different stages of differentiation [62]. Thus, it can be suggested that GATA6-AS1 plays a critical role in coordinating the expression of GATA6 across various tissues and cellular processes, highlighting the participation of lncRNAs in regulating stem cell fate decisions [27].

### 2.2. T-REX17

T-REX17 (Transcript Regulating Endoderm and activated by sox17) is the latest lncRNA identified in the context of endoderm differentiation of hiPSCs [55]. Differently from the above-described GATA6-AS1, T-REX17 is exclusively expressed in endoderm, with no detectable expression in mesoderm or ectoderm [55]. This novel lncRNA is transcribed only 230 kb away from the SOX17 gene and resides within the same topologically associated domain as this key TF [55], which contains a DNA-binding SRY-related high mobility group (HMG) box, common to all members of the SOX family [63]. Especially, SOX17 has been demonstrated to be necessary for specifying and maintaining definitive endoderm formation in vitro [64,65] and in vivo [66].

The presence of lncRNAs at different SOX gene loci has been reported, and most of them regulate the expression of their associated SOX genes in *cis* [67,68]. However, this is not true for T-REX17 and SOX17. Tracking T-REX17 during endoderm differentiation revealed that its expression follows the kinetics of SOX17 with a 24 h delay. Additionally, the expression of T-REX17 was uncoupled from that of SOX17 in different endoderm-derived tissues, with high specificity in the early stages of differentiation [55]. Orthogonal loss of function assays showed that T-REX17 did not affect the expression of SOX17 or impact its activation and regulation, making it dispensable for proper SOX17 regulation. Moreover, T-REX17 induction is dependent on SOX17 activation [55].

Regarding functional impacts on differentiation, T-REX17-depleted cells showed a decrease in the CXCR4^+^ cell population according to flow cytometry analysis, which led to impaired endoderm differentiation [55]. Moreover, the absence of T-REX17 also decreased the PDX1^+^ cell population on day 9 of direct differentiation to pancreatic progenitors, indicating that T-REX17 is not only essential for definitive endoderm formation but also impacts cell differentiation potential towards endoderm-derived tissues [55]. Thus, given that T-REX17 was essential for endoderm differentiation but does not regulate SOX17 in this process, it may imply a *trans*-acting role for this lncRNA in the endoderm [55] (Figure 1).

Interestingly, the authors identified an interaction between T-REX17 and several (hnRNPs) family members with exceptionally high levels of hnRNPU [55]. This suggests that lncRNA–ribonucleoprotein interactions may be one of the molecular mechanisms through which T-REX17 functions in endoderm differentiation [55]. The hnRNP family consists of more than 20 RBPs that are essential in binding nascent mRNA and regulating different biological processes, including transcription, alternative splicing, and protein translation [69]. The association with RBPs is a well-established mechanism through which lncRNAs exert their functions at the post-transcriptional level, as extensively reviewed elsewhere [7]. hnRNPU, in particular, was previously reported to interact with lncRNAs to regulate various functions during development, such as nuclear matrix organization for lncRNA Firre [70,71] and X chromosome activation for lncRNA XIST [72].

Although further studies are needed to elucidate the interaction between T-REX17 and hnRNPs fully, current evidence may link T-REX17-hnRNPs complex to a variety of nuclear functions responsible for lineage specification of hPSCs [55]. Moreover, T-REX17 depletion led to the upregulation of JUN pathway genes, while inhibition of JNK pathway hyperactivity partially rescued the endoderm differentiated phenotype of these cells [55], suggesting that T-REX17-hnRNPs may directly regulate the JUN pathway. Since most lncRNAs function alongside TFs in endoderm differentiation, as exemplified by GATA6-AS1 [44] and DEANR1 [46], the unraveling of T-REX17 provides an excellent opportunity to explore lncRNAs that operate independently and exert *trans*-acting regulatory mechanisms in this germ layer.

### 2.3. HIDEN

The analysis of genomic positions occupied by actively transcribed lncRNAs in hESCs illustrated that 90% are associated with promoters, enhancers, or bodies of protein-coding genes [73]. In endoderm, most of the studies available up until now, including the ones with GATA6-AS1 [44] and T-REX17 [55], focused on lncRNAs located physically close to lineage specification TFs (e.g., GATA6 and SOX17). However, the function of distal lncRNAs, particularly those located in gene deserts—referred to as desert lncRNAs—has been much less discussed due to challenges associated with large-scale screening, annotation and identification of the downstream target [54]. Therefore, whether and how desert lncRNAs far from protein-coding genes could regulate endoderm differentiation remained unexplored until recently.

HIDEN (human IMP1-associated “desert” definitive endoderm lncRNA) is the first functional desert lncRNA reported in human endoderm differentiation [54]. Identified through transcriptome analysis of hESCs, HIDEN resides more than 50 kb from any protein-coding gene and is gradually expressed during endoderm formation [54]. Compared to proximal lncRNAs that are usually genomically located in the nucleus, such as GATA6-AS1 [44] and T-REX17 [55], HIDEN was found to be mainly localized in the cytoplasmic fraction of endoderm cells, which implied a possible gene regulation at the post-transcriptional level by association with RBPs [54].

The interacting proteins of HIDEN were identified through RNA pull-down assay and this confirmed the presence of RBPs of the IMP (insulin-like growth factor 2 (IGF2) messenger RNA (mRNA)-binding proteins (IGF2BPs) family [54]. Highly conserved across species, each of the three members of the IMP family (IMP1, IMP2, and IMP3) contains six RNA-binding domains, including two RNA recognition motifs (RRM) and four K homology domains (KH) [74]. IMP1, for instance, was the most enriched protein physically interacting with HIDEN. Further structural analysis revealed that this interaction occurs between a part of HIDEN structure (251–650 nucleotides) and the KH3-4 domains of IMP1, which were directly responsible for the binding to the HIDEN sequence [54].

To further investigate the mechanism behind HIDEN–IMP1 interaction, IMP1-knockout cells were subjected to endoderm differentiation. IMP1 depletion resulted in impaired endoderm formation, and gene ontology analysis of top-downregulated genes showed enrichment related to endoderm specification, including regulation of the Wnt pathway [54]. HIDEN-knockout cells also exhibited defects in endoderm formation, and a comparison of gene ontology analysis of both knockout cell lines revealed 1051 genes co-regulated by HIDEN/IMP1, suggesting their participation in the same regulatory loop [54]. As the downregulation of the WNT/β-catenin pathway was observed in both knockout cell models, the protein levels of active β-catenin were measured. Loss of HIDEN or IMP1 decreased β-catenin expression and further impaired WNT activity, leading to defects in endoderm [54].

The WNT/β-catenin complex comprises 19 different WNT proteins, 10 frizzled (FZD) receptors, and a variety of other co-receptors [75]. The FZD receptors are part of the superfamily of G protein-coupled receptors (GPCR), with an N-terminus containing a cysteine-rich domain (CRD) responsible for FZD binding WNT ligands and a C-terminus that interacts with G protein through binding to Dishevelled (Dvl) [76]. One of the FZD receptors, FZD5, was found to be differentially expressed both in HIDEN-knockout cells and IMP1-bound genes in RIP-seq analysis of endoderm cells, suggesting it as a potential direct target of HIDEN/IMP1 [54]. Functional analysis further revealed that FZD5 expression is upregulated in endoderm differentiation but is severely reduced upon HIDEN depletion. Moreover, the interaction between IMP1 and FZD5 mRNA was dependent on HIDEN, as lower levels of HIDEN in knockout cells impaired the binding of IMP1 to FZD5 and decreased its expression, reducing endoderm differentiation [54]. Treatment with Actinomycin D resulted in destabilization of FZD5 mRNA in HIDEN-knockout endoderm cells and, upon IMP1 knockout, a decrease in FZD5 expression, indicating the role of HIDEN in promoting the stability of FZD5 mRNA via IMP1 [54]. Thus, lncRNA HIDEN interacts with the KH3-4 domains of IMP1 protein and enhances FZD5 stability by facilitating the interaction between IMP1 and FZD5 mRNA [54] (Figure 1).

Other FZD receptors, such as FZD7, have been linked to hESC self-renewal and differentiation [77,78]. In this case, FZD7 was required for maintaining the pluripotent state of hESCs [77], while its selective activation was demonstrated to promote meso-endodermal differentiation of hiPSCs [78]. The mechanistic evaluation of HIDEN further enhances understanding the role of FDZ family receptors and the WNT signaling pathway in endoderm differentiation. Moreover, recent studies showed that IMP1 binds to mRNAs, facilitating the formation of messenger ribonucleoprotein RNP granules in the cytoplasm [79,80], regulating homeostasis and protecting target mRNAs from miRNA-mediated silencing or premature release to translation [80]. Whether the involvement of HIDEN in FDZ5 stabilization by IMP1 is related to RNP granule formation remains unclear and more studies are needed to clarify the structural basis of how lncRNAs participate in IMP1-regulated mRNA stability [54]. Nevertheless, HIDEN discovery and characterization provide new insights into post-transcriptional regulation mediated by lncRNAs. Additionally, it opens new avenues for exploring the function of desert lncRNAs in cell fate and lineage specification of stem cells.

## 3. Conclusions and Future Directions

The molecular functions of lncRNAs play a crucial role in deciphering the regulatory networks that govern biological processes. As one of the most structurally and functionally diverse classes of ncRNAs, lncRNAs regulate gene expression at transcriptional, post-transcriptional, and epigenetic levels [7]. Despite growing interest, endoderm-associated lncRNAs have only recently begun to be explored, and their mechanisms of action remain largely unknown [55]. This review examined the role of lncRNAs in human endoderm differentiation, emphasizing their distinct regulatory functions in this process (Figure 1). GATA6-AS1 has been identified as a transcriptional regulator of endoderm differentiation [44], while HIDEN functions at the post-transcriptional level [54]. Although the precise regulatory mechanism by which T-REX17 modulates endoderm formation is not yet fully understood, evidence suggests it regulates the JUN pathway [55].

Functional characterization of lncRNAs encounters multiple limitations and methodological challenges [81]. First, annotating lncRNAs from transcriptomic data is difficult due to their low expression, species-specificity, and space–temporal expression patterns [81]. Second, predicting structural 2D and 3D domains is not always possible as many lncRNAs do not have readily identifiable open reading frames (ORFs), making it challenging to correlate lncRNA sequence to their function [82]. Conversely, recent studies have revealed that some transcripts previously annotated as lncRNAs contain small open reading frames (sORFs) that are actively translated into micropeptides [83], such as DEANR1 in endoderm [47]. In this case, the micropeptides and the RNA transcript may exert different functions in the same biological process, adding a layer of complexity to the functional characterization of lncRNAs [83]. Lastly, low sequence conservation of lncRNAs across species complicates the extraction of information from comparative evolutionary analyses [84].

However, one of the major challenges lies in the functional plasticity of lncRNAs in biological processes [27]. The ability to regulate gene expression at multiple levels in different contexts adds further complexity in predicting biological functions of novel lncRNAs [27]. A single lncRNA can employ multiple regulatory mechanisms simultaneously within the same process, as seen with DIGIT, which functions both in *cis* and *trans* during endoderm differentiation [48,49]. In this sense, integrating multiple methodological approaches is essential for studying lncRNA mechanisms, each of which presents limitations [81]. For example, lncRNA knockout via CRISPR-Cas9-mediated locus deletion provides stable cell effects but may disrupt regulatory elements within the genomic locus, while lncRNA knockout by promoter deletion may alter the regulatory DNA sequence, affecting neighboring genes [81]. For instance, CRISPR interference (CRISPRi) is used to assess lncRNA transcription initiation, with knockdown efficiency depending on the specific locus target. Knockdown of lncRNAs can also be achieved using RNA interference (RNAi), which is particularly appealing for cytoplasmatic lncRNAs. However, its transient effect and the potential of off-target effects should be carefully considered [81]. Thus, integrating high-throughput screening and transcriptomic analyses, functional assays, and in vivo and novel in vitro models will be essential to fully characterize the role of lncRNAs in developmental stem cell research, including endodermal lineage commitment and differentiation. A deeper exploration of lncRNA mechanisms in endoderm could provide valuable insights into both the regulatory aspects of the normal differentiation process and the formation of endoderm-derived tissues, such as the liver and pancreas, with significant implications for regenerative medicine as well.

## Figures and Tables

**Table 1:** Endoderm-associated lncRNAs and their mechanism of action.

lncRNA	Subcellular Localization	Mechanism of Action	Phenotype
DEANR1 [46]	Nuclear	*Cis*-regulation by transcriptional activation of FOXA2	Promotes differentiation
DIGIT [48,49]	Nuclear	*Trans*-regulation of GSC by interaction with BRD3 protein at sites of H3K18ac	Promotes differentiation
Gas5 [50]	Nuclear	Not described in endoderm	Represses differentiation in mESCs
GATA6-AS1 [44]	Nuclear	*Cis*-regulation by facilitating GATA6 transcriptional activation through binding to SMAD2/3	Promotes differentiation
LINC00458 [52]	Nuclear	*Trans*-regulation by interaction with SMAD2/3 to modulate soft substrate-induced endoderm differentiation	Promotes differentiation
HOXA-AS3 [53]	Nuclear	*Cis*-regulation by facilitating chromatin accessibility	Promotes differentiation
HOXB-AS3 [53]	Nuclear	*Cis*-regulation by facilitating chromatin accessibility	Promotes differentiation
HIDEN [54]	Cytoplasmatic	*Trans*-regulation by physically interacting with IMP1 to facilitate mRNA stabilization of FZD5	Promotes differentiation
T-REX17 [55]	Nuclear	Possible *trans*-regulation by interaction with heterogeneous ribonucleoproteins (hnRNPs)	Promotes differentiation

## Abbreviations

The following abbreviations are used in this manuscript:

ncRNAs	Non-coding RNAs
lncRNAs	Long non-coding RNAs
miRNAs	Micro-RNAs
mRNAs	Messenger RNAs
UTRs	Untranslated regions
TFs	Transcription factors
RBPs	RNA-binding proteins
RNP	Ribonucleoprotein
PRC2	Polycomb repressive complex 2
hnRNPs	Heterogeneous nuclear ribonucleoproteins
hPSCs	Human pluripotent stem cells
hiPSCs	Human induced pluripotent stem cells
hESCs	Human embryonic stem cells
mESCs	Mouse embryonic stem cells
ceRNA	Competitive endogenous RNA
PS	Primitive streak
FZD5	Frizzled 5
*HMG*	High mobility group
*GATA6-AS1*	GATA6 antisense RNA 1
*T-REX17*	Transcript regulating endoderm activated by SOX17
*HIDEN*	Human IMP1-associated desert definitive endoderm lncRNA
IMP	Insulin-like growth factor 2 messenger RNA-binding proteins
RRM	RNA recognition motifs
KH	K homology domains
CRD	Cysteine-rich domain
GPCR	G protein-coupled receptors
Dvl	Dishevelled
ORFs	Open reading frames
sORFs	Smal open reading frames
CRISPRi	CRISPR interference
RNAi	RNA interference
